# Rapid Detection of Lipopolysaccharide and Whole Cells of *Francisella tularensis* Based on Agglutination of Antibody-Coated Gold Nanoparticles and Colorimetric Registration

**DOI:** 10.3390/mi13122194

**Published:** 2022-12-11

**Authors:** Nadezhda A. Byzova, Anatoly V. Zherdev, Alexey A. Gorbatov, Anton G. Shevyakov, Sergey F. Biketov, Boris B. Dzantiev

**Affiliations:** 1 A.N. Bach Institute of Biochemistry, Research Center of Biotechnology, Russian Academy of Sciences, 119071 Moscow, Russia; 2 State Research Center for Applied Microbiology and Biotechnology, 142279 Obolensk, Russia

**Keywords:** immunoanalysis, agglutination, colloidal gold, tularemia, lipopolysaccharide, express diagnostics

## Abstract

The paper presents development and characterization of a new bioanalytical test system for rapid detection of lipopolysaccharide (LPS) and whole cells of *Francisella tularensis*, a causative agent of tularemia, in water samples. Gold nanoparticles (AuNPs) coated by the obtained anti-LPS monoclonal antibodies were used for the assay. Their contact with antigen in tested samples leads to aggregation with a shift of absorption spectra from red to blue. Photometric measurements at 530 nm indicated the analyte presence. Three preparations of AuNPs with different diameters were compared, and the AuNPs having average diameter of 34 nm were found to be optimal. The assay is implemented in 20 min and is characterized by detection limits equal to 40 ng/mL for LPS and 3 × 10^4^ CFU/mL for whole cells of *F. tularensis*. Thus, the proposed simple one-step assay integrates sensitivity comparable with other immunoassay of microorganisms and rapidity. Selectivity of the assay for different strains of *F. tularensis* was tested and the possibility to choose its variants with the use of different antibodies to distinguish virulent and non-virulent strains or to detect both kinds of *F. tularensis* was found. The test system has been successfully implemented to reveal the analyte in natural and tap water samples without the loss of sensitivity.

## 1. Introduction

In recent years, colorimetric biosensors have attracted wide attention due to their simplicity and low cost. Colorimetric biosensors usually do not require complex expensive equipment because color changes can be detected with the naked eye. Therefore, they can be used for field analysis and on-site diagnostics [1]. The change of color can be promoted by specific interactions of gold, silver, and other metal nanoparticles, including those resulting in their aggregation [2,3]. Among the advantages of gold nanoparticles (AuNPs), stability, easy complexation to various biomolecules, and variable optical properties can be mentioned, which make them an ideal marker for colorimetric biosensors [4]. The absorption at 520–530 nm (red color) corresponds to small AuNPs while the shift of the absorption maximum to the longer wavelength region (550 nm and more, blue and violet colors) refers to aggregated AuNPs [5]. Mechanisms of such shifts have been analyzed; their high reproducibility allows for the implementation of reliable qualitative and quantitative assays [5].

Among various analytes, cells of microorganisms cause significant interest due to the possibility of their simple and rapid detection [6,7,8]. AuNPs with immobilized antibodies, aptamers, lectins, bacteriophages, and other receptors were successfully applied for the selective detection of different microorganisms. Depending on the assay format, microorganisms can be detected either by the aggregation of AuNPs or by the dissociation of AuNPs–receptor complexes. Thus, salt-induced aggregation of AuNPs functionalized by 4-mercaptophenylboronic acid was used to detect *Escherichia coli* in drinking water [9]. AuNPs–aptamer complexes dissociated in the presence of *Shigella flexneri* with the following aggregation of destabilized free AuNPs and a change of their color from red to violet [10]. The complexes of thiolated bacteriophages with *Pseudomonas aeruginosa*, *Vibrio cholerae*, and *Xanthomonas campestris* initiated binding with AuNPs followed by visible color change [11]. Simultaneous immobilization of antibodies and Concanavalin A on the surface of magnetite particles was applied for aggregation-based detection of *Escherichia coli* cells [12]. Rapid detection of the influenza A virus was performed using antibody-functionalized AuNPs. Dynamic light scattering (DLS) was applied for the detection of the formation of large aggregates accompanied by color change [13]. The given developments allow selective detection of target pathogens but do not provide detailed comparative evaluation of different assay variants. Therefore, the questions of the most efficient gold nanoparticles preparations, as well as conditions of immune interactions require new choices when developing aggregation test systems for new analytes.

However, despite the easy colorimetric registration of changes in the state of AuNPs, it is used less frequently compared to the dominant latex agglutination tests. Recent publications on the application of AuNPs as analytical reactants (see reviews [2,14] summarizing variety of the existing developments) describe numerous sophisticated assay formats. At the same time, for the simplest analysis based on color changes initiated by the complexation of bacteria and AuNPs functionalized with receptor molecules (i.e., antibodies), some issues needs additional study and understanding, as recent reviews about bioanalytical possibilities of AuNPs demonstrated [5,15,16]. For example, the choice of particles’ size and interaction conditions is not clear and newly considered for each case. Although rapid analytical methods are needed to detect disease-causing microorganisms in the environment and in samples from patients, simple approaches based on the aggregation of AuNPs are not developed and characterized as tools for the detection of many of the pathogenic microorganisms.

Tularemia is a zoonotic infection caused by the gram-negative bacterium *Francisella tularensis*. Four subspecies are distinguished within the *F. tularensis*—*tularensis*, *holarctica*, *mediasiatica*, and *novicida* [17,18]. The strains of the subspecies *tularensis* have the highest virulence for humans while the strains of the subspecies *holarctica* and *mediasiatica* are less virulent. *F. tularensis* subsp. *novicida* is considered an opportunistic pathogen for humans [19]. High susceptibility for humans and significant mortality (26.8%) in the absence of countermeasures is the reason for considering tularemia as a particularly dangerous infection [20,21]. Significant risks to human health due to the frequent occurrence of the tularemia pathogen in environmental objects necessitate extensive monitoring of contamination, which requires simple and fast analytical methods [22,23].

Currently, various methods are used for the clinical diagnosis of tularemia. Among them, isolating a pure culture of the pathogen from biosamples [24,25], molecular genetic methods such as polymerase chain reaction [26,27], immunological methods (the enzyme-linked immunosorbent assay (ELISA), immunochromatographic assay (ICA), and Western blot) [28,29], as well as serological methods that detect specific antibodies to the causative agent of tularemia in the sera of patients [30,31,32,33] can be mentioned. The limit of detection (LOD) of *F. tularensis* cells is 10^3^–10^4^ CFU/mL [28] and 10^5^–10^8^ CFU/mL [28,30] in the ELISA and ICA, respectively. Among the techniques for tularemia diagnosis presented in this paragraph, only ICA allows for rapid testing, while the other methods considered above require specialized equipment to obtain results; their duration is 3–18 h. The ICA can be completed within 15–20 min excluding the sample preparation but its sensitivity is inferior to the ELISA by two orders of magnitude [28]. Therefore, new developments of methods for immunodetection of the causative agent of tularemia are in demand, which are characterized by rapid and simple implementation and acceptable sensitivity to successfully compete with the existing methods.

The indicated above limitations of common instrumental immunoassays such as ELISA in duration and rapid immune tests such as ICA in sensitivity determines the demand for simple non-instrumental methods for tularemia diagnostics, which are necessary for making a preliminary diagnosis and conducting epidemiological monitoring in endemic areas. The presented study was focused on the development and characterization of a rapid assay based on conjugates of AuNPs with antibodies to the *F. tularensis* antigen (lipopolysaccharide, LPS) and to determine its analytical performance. The proposed simple one-step assay allows detecting up to 40 ng/mL of LPS and 3 × 10^4^ CFU/mL of *F. tularensis* cells in 20 min, thus integrating rapidity and sensitivity and demonstrating competitive potential for its further consideration as tool for wide environmental screening of the presence of *F. tularensis* and risks of tularemia infection.

## 2. Materials and Methods

### 2.1. Materials and Chemicals

Sephadex G-25, protein-A-sepharose (GE Healthcare, Chicago, IL, USA), and culture medium RPMI-1640 (Thermo Fisher Scientific, Waltham, MA, USA) were used in the study. Fetal bovine serum was purchased from Cytiva (Marlborough, MA, USA). Gold (III) chloride (HAuCl_4_), sodium citrate, sodium azide, bovine serum albumin (BSA), sucrose, Tris, Tween-20, Triton X-100, 3,3′,5,5′-tetramethylbenzidine dihydrochloride (TMB), pristane, polymyxin B, thimerosal, ethidium bromide, DNAase, RNAse, and proteinase K were from Sigma-Aldrich (St. Louis, MO, USA). Goat antibodies against mouse immunoglobulins labeled with horseradish peroxidase (GAMI–HRP, Jackson ImmunoResearch, Cambridgeshire, UK) were used. Heterologous strains of microorganisms (*Yersinia enterocolitica* H-26-04 and 287, *Y. pseudotuberculosis* 4320, *Pseudomonas aeruginosa* ATCC27853, *Brucella abortus*, *Salmonella* spp.) were obtained from the State Collection of Pathogenic Microorganisms and Cell Cultures “GKPM–OBOLENSK” FBSI SRC PMB (Obolensk, Moscow region, Russia). BALB/c mice were from the Federal Scientific Center for Biomedical Technologies of the Federal Medical and Biological Agency (Stolbovaya, Moscow region, Russia).

All other chemicals were purchased from Himmed (Moscow, Russia); they were of analytical grade and were used without purification. All solutions were prepared with ultrapure water with a resistivity of 18.2 MW (Millipore Corporation, Burlington, MA, USA). The 96-well transparent polystyrene microplates for the ELISA were purchased from Corning Costar (Tewksbury, MA, USA). Samples of natural water were taken from a river, lake, and spring located in the Moscow region.

### 2.2. Bacterial Strains, Cultivation, and Inactivation Conditions

*F. tularensis* strains (*F. tularensis* subsp. *holarctica* 15 NIIEG, 503, and *miura*, *F. tularensis* subsp. *tularensis* Schu and A-Cole B-399, *F. tularensis* subsp. *mediasiatica* 120, and *F. tularensis* subsp. *novicida* Utah112) were cultured for 24 h at 37 °C on solid nutrient medium FT-agar with black albumin with the addition of polymyxin B to a concentration of 100 mg/L.

The grown cells were suspended in 0.15 M NaCl to a concentration of 5 × 10^9^ CFU/mL. For thermal inactivation, the suspension was incubated for 1 h at 70 °C. For chemical inactivation, the suspension was incubated for 24 h at 20–25 °C in the presence of 0.1% thimerosal. Inactivated cells were tested for sterility by plating them on thioglycol medium and FT agar.

The *F. tularensis* subsp. *holarctica* 15 NIIEG strain was used to obtain LPS (see supplementary information).

### 2.3. Synthesis of AuNPs

Three preparations of spherical AuNPs with expected average diameters of 20–30, 30–40, and 40–50 nm were synthesized using the citrate method of Frens [34] with some modifications [35]. For this purpose, sodium citrate (1.75, 1.5, and 1.0 mL of 1% solution) was added to 100 mL of boiling 0.01% solution of HAuCl_4_ under stirring. The mixtures were boiled for 25 min. The obtained AuNPs were cooled and stored at 4–6 °C. The resulting preparations were designated as AuNPs1, AuNPs2, and AuNPs3.

### 2.4. Characterization of AuNPs

The absorption spectra of AuNPs1, AuNPs2, and AuNPs3 were recorded on a Biochrom Libra S80 spectrophotometer (Biochrom, Cambridge, UK) in the wavelength range of 400–700 nm. The dimensions and homogeneity of AuNPs1, AuNPs2, and AuNPs3 were characterized by transmission electron microscopy (TEM) as described previously [36]. AuNPs were applied to grids (300 mesh) coated with a film of polyvinyl formal dissolved in chloroform. The preparations were observed on a JEM CX-100 electron microscope (JEOL, Tokyo, Japan) at an accelerating voltage of 80 kV and a magnification of 3,300,000. The photographs were digitally analyzed using the Image Tool program (University of Texas Health Science Center, San Antonio, TX, USA).

The hydrodynamic size of AuNPs was measured using a Zetasizer Nano (Malvern Pananlytical, Malvern, UK) as described previously [36]. DLS was registered at 25 °C for 10 s at a scattering angle of 173°. Each sample was analyzed by three 30-second cycles.

### 2.5. Synthesis and Characterization of AuNPs–MAbs Conjugates

Conjugates of monoclonal antibodies (MAbs) against *F. tularensis* LPS (clones Fb11 and T143) with AuNPs1, AuNPs2, and AuNPs3 were synthesized by adsorption immobilization (production of MAbs—see supplementary information). Before conjugation with AuNPs, MAbs was dialyzed against a 1000-fold volume of 10 mM Tris-HCl buffer, pH 9.0, for 2 h at 4–6 °C. AuNPs1, AuNPs2, and AuNPs3 (OD = 1) with pH adjusted to 9.0 by 0.1 M K_2_CO_3_ were added to MAbs solutions (pH 9.0) and incubated for 30 min at 20–22 °C with stirring. Then, an aqueous solution of BSA was added to the mixtures to a final concentration of 0.25%. After that, AuNPs–MAbs conjugates were separated using an Allegra 64R centrifuge (Beckman Coulter, Indianapolis, IN, USA) for 15 min at 4 °C at 22,000× *g* (for AuNPs1–MAbs), 18,000× *g* (for AuNPs2–MAbs) and 15,000× *g* (for AuNPs3–MAbs). The precipitates were resuspended in 0.01 M Tris-HCl, pH 9.0, containing 1.0% BSA, 1.0% sucrose, and 0.01% sodium azide and stored at 4–6 °C.

Spectral characterization and determination of the hydrodynamic diameters of the synthesized conjugates were performed as described in the previous section for AuNPs.

### 2.6. Characterization of AuNPs–MAbs Conjugates by ELISA

Functional characterization of AuNPs–MAbs conjugates was performed by the ELISA. The *F. tularensis* LPS antigen (3 µg/mL, 100 µL) in 50 mM potassium phosphate buffer, pH 7.4, containing 0.1M NaCl (PBS) was immobilized in microplate wells overnight at 4 °C. Then, the microplate was washed four times with PBS containing 0.05% Triton X-100 (PBST). Next, solutions of Fb11 and T143 clones in PBST (concentration range from 0.01 to 10 μg/mL, 100 μL) and AuNPs–Fb11 and AuNPs–T143 conjugates in PBS (OD range from 0.0001 to 2, 100 μL) were added to the microplate wells. The microplate was incubated for 1 h at 37 °C and washed four times. After that, GAMI–HRP in PBST (1:5,000 dilution, 100 µL) was added to the wells and incubated again for 1 h at 37 °C. After washing, the activity of the HRP label was determined. For this, 100 μL of a 0.4 mM solution of TMB in 40 mM sodium citrate buffer, pH 4.0, containing 3 mM hydrogen peroxide, was added to the microplate wells and incubated for 15 min at room temperature. The reaction was stopped by adding 50 µL of 1 M sulfuric acid, and OD_450_ was measured by Zenyth 3100 microplate spectrophotometer (Anthos Labtec Instruments, Salzburg, Austria).

### 2.7. Agglutination of AuNPs–MAbs Conjugates in the Presence of F. tularensis LPS and Cells

Solutions of *F. tularensis* LPS and cells in PBST (concentration range from 30 μg/mL to 10 ng/mL and from 10^9^ to 10^3^ CFU/mL, respectively) were prepared. LPS and cell solutions (75 μL both) were added to the microplate wells. Then, AuNPs–MAbs conjugates (OD = 2; 75 µL) were added to each well and incubated for 1, 10, 20, and 30 min at room temperature. When testing samples of natural and tap water, solutions of *F. tularensis* cells and the conjugate were prepared in water containing 0.05% Triton X-100 to prevent nonspecific binding. Adsorption spectra were recorded on a Perkin Elmer En Spire 2300 microplate reader (Waltham, MA, USA) in the wavelength range of 500–600 nm. Three measurements were performed for each antigen concentration.

## 3. Results and Discussion

### 3.1. Synthesis and Characterization of AuNPs

Smaller AuNPs are known to form more stable colloidal solutions but larger AuNPs may be more sensitive in agglutination-based assays of microorganisms [37]. Therefore, when developing an agglutination test system, it was important to optimize the size of nanoparticles. AuNPs were synthesized by the modified Frens method using sodium citrate as a reducing agent in concentrations of 0.6, 0.51, and 0.34 mM to obtain AuNPs with the expected average diameters of 20–30, 30–40, and 40–50 nm, respectively. The resulting AuNPs were characterized by TEM(JEOL, Tokyo, Japan; University of Texas Health Science Center, San Antonio, TX, USA), DLS (Malvern Pananlytical, Malvern, UK), and spectrophotometry (Biochrom, Cambridge, UK).

**Figure 1:**
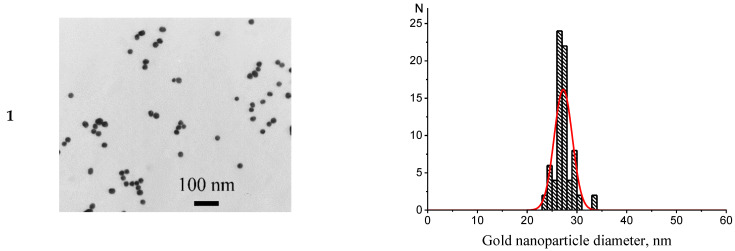

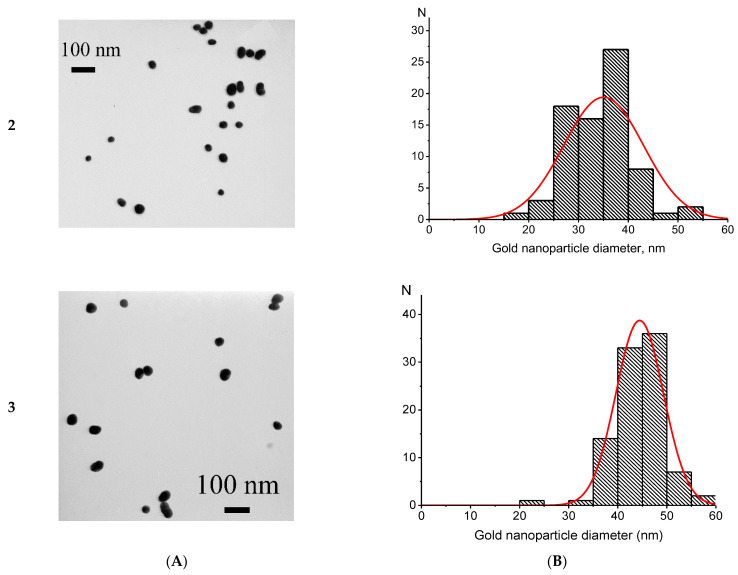
Electron microphotographs (**A**) of AuNPs1 (**1**), AuNPs2 (**2**), and AuNPs3 (**3**) and histograms of diameter distribution (**B**).

Figure 1 presents the results of the characterization of AuNPs1, AuNPs2, and AuNPs3 by TEM. As can be seen, all preparations did not contain aggregates and the particle shape was close to spherical. According to TEM images, the average diameters/ellipticity of AuNPs were 26.6 ± 1.6 nm/1.1 ± 0.14 (AuNPs1), 33.7 ± 2.3 nm/1.2 ± 0.17 (AuNPs2), and 41.8 ± 4.2 nm/1.3 ± 0.27 (AuNPs3).

The hydrodynamic dimensions of AuNPs were determined by the DLS as described in [36]; the results are presented in Figure S1. The synthesized AuNPs1, AuNPs2, and AuNPs3 had hydrodynamic diameters/polydispersity indices (Pdi) of 26.8 nm/0.236, 36.8 nm/0.266, and 41.7 nm/0.286, respectively, indicating the stability of colloids (Pdi < 0.3). The synthesized AuNPs were also characterized spectrophotometrically. Spherical AuNPs have a characteristic absorption peak at wavelengths of 515–540 nm, which is associated with the surface plasmon resonance between the frequency of collective oscillations of free electrons on the AuNPs surface and the frequency of the light wave [38,39]. With the increase of AuNPs’ diameter, their absorption spectra shifted to longer wavelengths (523, 526, and 530 nm for AuNPs1, AuNPs2, and AuNPs3, respectively) (Figure S2). The size of AuNPs was estimated using the earlier obtained dependence of the absorption spectrum maximum on AuNPs’ diameter [40]. For AuNPs1, AuNPs2, and AuNPs3, the average diameters were 26, 34, and 42 nm, respectively. Table 1 shows the dimensional characteristics of AuNPs.

The dimensional characteristics of AuNPs obtained by the three techniques are in good agreement with each other. The differences are no more than 3.1% for AuNPs1, 8.2% for AuNPs2, and 0.7% for AuNPs3.

### 3.2. Synthesis and Characterization of AuNPs–MAbs Conjugates

After studying the properties of native AuNPs, their conjugates with MAbs against *F. tularensis* LPS (clones Fb11 and T143) were obtained by adsorption immobilization. MAbs concentrations were calculated based on the size of the protein globule and the average surface area occupied by antibodies during monolayer immobilization (25 nm^2^) according to the method described earlier [40]. MAbs concentrations for monolayer immobilization on the surface of AuNPs1, AuNPs2, and AuNPs3 are given in Table 2.

During monolayer immobilization of MAbs on the surface of AuNPs with average diameters of 26, 34, and 42 nm, only 43, 35, and 25% of MAbs added during the synthesis become immobilized [40]. The modes of conjugate sedimentation by centrifugation were selected based on the previously obtained results for AuNPs having diameters of 20–50 nm [36]. Thus, 6 preparations of AuNPs–MAbs conjugates with equal loading of MAbs on AuNPs’ surface were obtained, namely, AuNPs1–Fb11, AuNPs2–Fb11, AuNPs3–Fb11, AuNPs1–T143, AuNPs2–T143, and AuNPs3–T143. The spectrophotometric characterization of the synthesized AuNPs–MAbs conjugates carried out under the conditions described in the previous section showed that the maxima of the absorption spectra of the conjugates were shifted to the long wavelength region compared to unloaded AuNPs. It corresponded to an increase in the average diameter by 6–8 nm (Table 3).

According to the DLS data (Figures S3 and S4), the average diameters of AuNPs–Fb11conjugates increased more than those of AuNPs–T143 conjugates (by 9.9–15.8 and 3.5–8.8 nm, respectively), which indicated lower stability of AuNPs–Fb11 conjugates. This is also confirmed by the Pdi of the AuNPs–Fb11 conjugates, which were 0.421–0.478 (values more than 0.3 indicate the conjugates’ instability).

**Figure 2:**
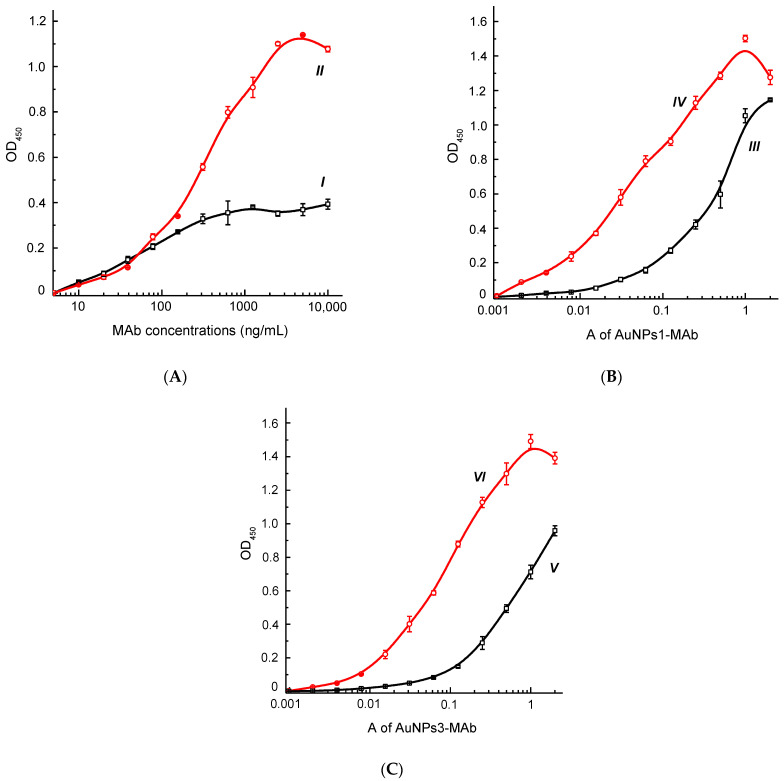
Dependences of OD_450_ on the concentration of MAbs (**A**) of clones Fb11 (I) and T143 (II) and the OD of AuNPs1–Fb11 ((**B**), III), AuNPs1–T143 ((**B**), IV), AuNPs3–Fb11 ((**C**), V), and AuNPs3–T143 ((**C**), VI) conjugates. *F. tularensis* LPS was immobilized in the microplate wells at the concentration of 3 µg/mL. All measurements were performed in triplicate.

Comparative functional characterization of AuNPs–MAbs conjugates was carried out by binding with *F. tularensis* LPS immobilized in microplate wells during the ELISA. Figure 2 shows the concentration dependences of the binding of specific MAbs (Figure 2A), AuNPs1–MAbs conjugate (Figure 2B), and AuNPs3–MAbs conjugate (Figure 2C) with *F. tularensis* LPS. It should be noted that the high titer of conjugates in the ELISA does not guarantee the maximum detection sensitivity in other assay formats. Therefore, the final choice of conjugates was made in the agglutination analysis.

### 3.3. Agglutination of AuNPs–MAbs Conjugates in the Presence of F. tularensis LPS

Agglutination of AuNPs–MAbs conjugates in the presence of *F. tularensis* LPS was performed by mixing solutions with different concentrations of antigens and a fixed concentration of AuNPs–MAbs conjugates. Adsorption spectra were recorded in the wavelength range from 500–600 nm. Figures S5 and S6 show the agglutination spectra of AuNPs2–Fb11 and AuNPs2–T143 conjugates in the presence of *F. tularensis* LPS at concentrations from 30 to 0.03 μg/mL. For the AuNPs2–Fb11 conjugate, the maximum changes in the agglutination spectra occur upon the addition of 10 μg/mL of LPS (Figure S5), and for the conjugates with MAbs T143—of 0.3 μg/mL (Figure S6).

**Figure 3:**
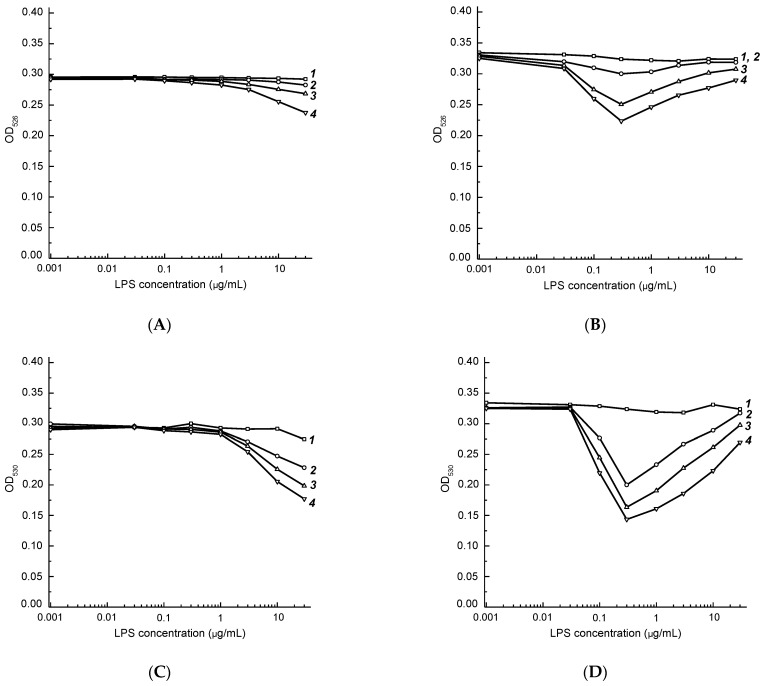

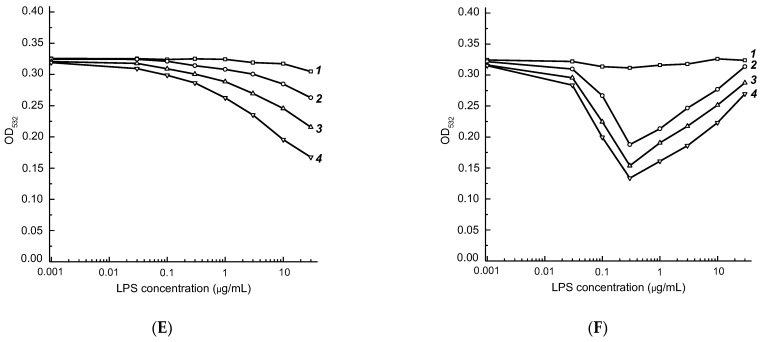
Agglutination of AuNPs1–Fb11 (**A**), AuNPs1–T143 (**B**), AuNPs2–Fb11 (**C**), AuNPs2–T143 (**D**), AuNPs3–Fb11 (**E**), and AuNPs3–T143 (**F**) conjugates in the presence of various concentrations of *F. tularensis* LPS. Curves 1–4 correspond to 1, 10, 20, and 30 min of the reaction.

To compare conjugates of MAbs with AuNPs1, AuNPs2, and AuNPs3 in terms of their agglutination ability and to select the optimal size of AuNPs, the concentration dependences for LPS were recorded at fixed wavelengths of absorption maxima: 526 nm for AuNPs1, 530 nm for AuNPs2, and 532 nm for AuNPs3 (Table 3). The corresponding concentration dependences are shown in Figure 3.

The shape of the agglutination curves in Figure 3B,D,F with a minimum at LPS concentrations of 0.1–1 μg/mL can be explained by the formation of large branched immune complexes, which are formed at equal molar concentrations of antibodies and polyvalent antigens (“equivalence zones”) and cause a change in the color of the reaction mixture from red to violet (Figure S7) [41].

The optimal time of agglutination was chosen based on OD changes at 526 nm (AuNPs1), 530 nm (AuNPs2), and 523 nm (AuNPs3), and LPS concentrations of 10 and 0.3 μg/mL for the AuNPs–Fb11 and AuNPs–T143 conjugates, respectively (Table 4). Within 10 min, the OD decreases by 17.4–47.7% for conjugates with the clone Fb11 and by 23.5–69.5% for conjugates with clone T143. Within 20 min, the OD decreases by 43.0–77.9% for conjugates with the clone Fb11 and by 73.0–88.9% for conjugates with the clone T143. Therefore, 20-minutes duration is sufficient for color changes and, consequently, the ability to detect the assay results visually and spectrophotometrically. As a result, AuNPs2–Fb11 and AuNPs2–T143 conjugates and the reaction time of 20 min were chosen for further experiments.

### 3.4. Agglutination of AuNPs–MAbs Conjugates in the Presence of F. tularensis Cells

**Figure 4:**
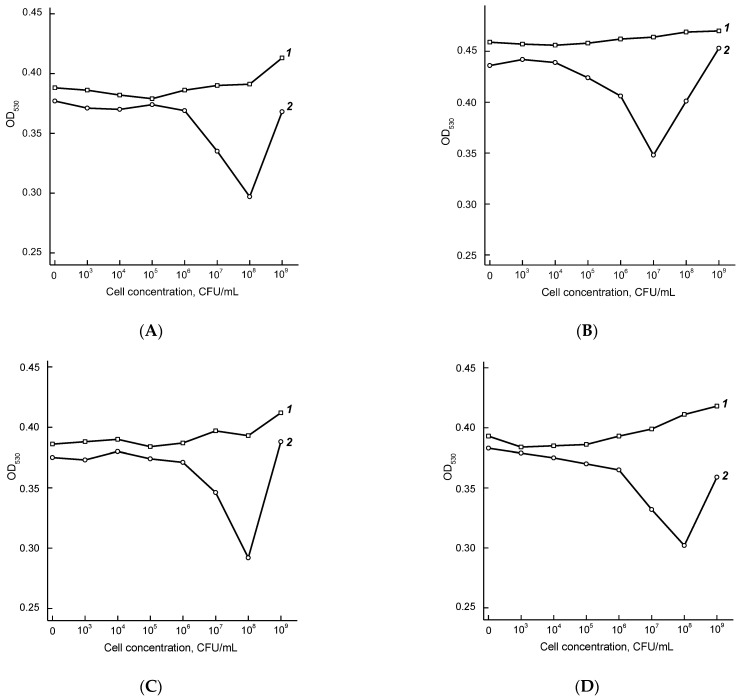

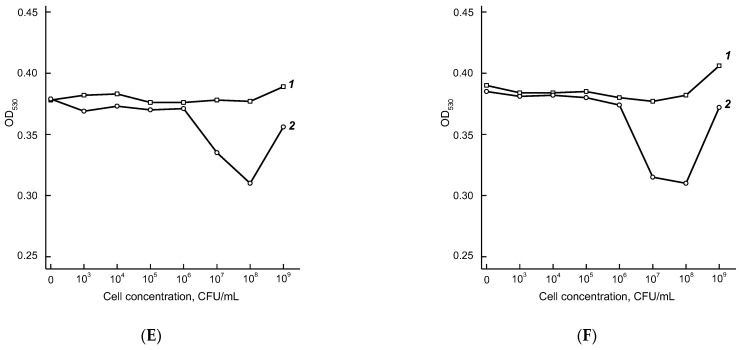
Agglutination of AuNPs2–Fb11 conjugate in the presence of various concentrations of thermally inactivated *F. tularensis* subsp. *holarctica 15 NIIEG* (**A**) and 503 (**B**), *F. tularensis* subsp. *mediasiatica 120* (**C**), *F. tularensis* subsp. *tularensis Schu* (**D**) and *A-Cole B-399* (**E**), and *F. tularensis* subsp. *novicida Utah112* (**F**). Curves 1 and 2 correspond to 1 and 20 min of the reaction.

**Figure 5:**
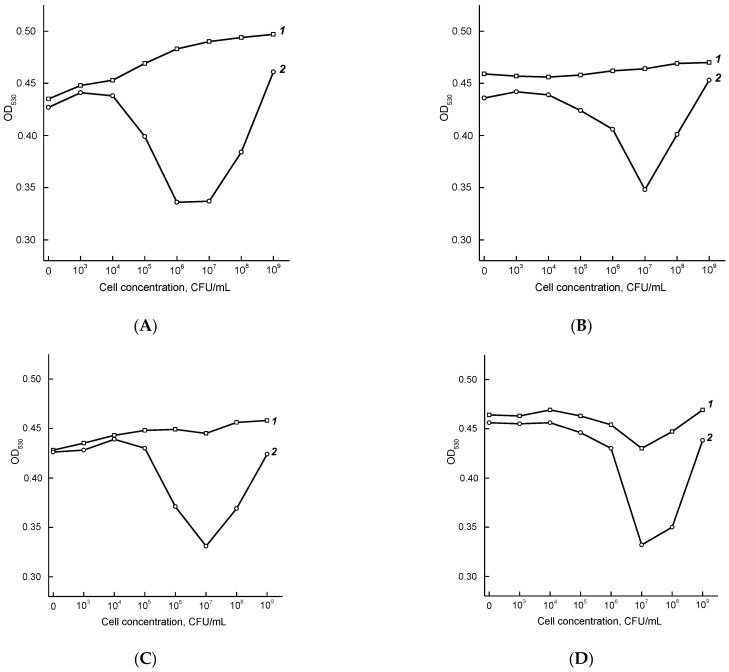

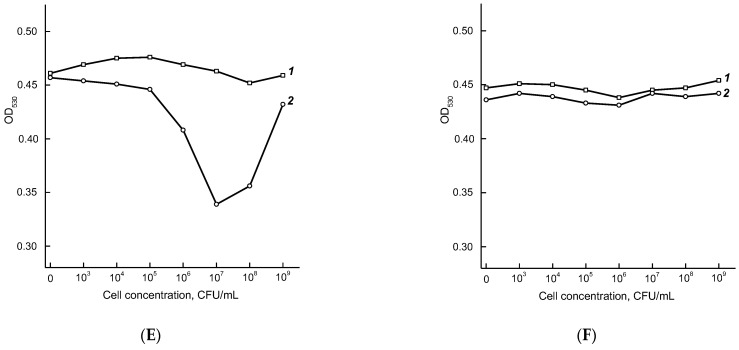
Agglutination of AuNPs2–T143 conjugates in the presence of various concentrations of thermally inactivated *F. tularensis* subsp. *holarctica 15 NIIEG* (**A**) and *503* (**B**), *F. tularensis* subsp. *mediasiatica 120* (**C**), *F. tularensis* subsp. *tularensis Schu* (**D**) and *A-Cole B-399* (**E**), and *F. tularensis* subsp. *novicida Utah112* (**F**). Curves 1 and 2 correspond to 1 and 20 min of reaction.

Agglutination of the AuNPs2–Fb11 and AuNPs2–T14 conjugates in the presence of *F. tularensis* cells was performed as described above. The cell concentration varied from 10^9^ to 10^4^ CFU/mL. Figure 4 and Figure 5 show the dependences of OD on the concentration of thermally inactivated *F. tularensis* cells, and Figures S8 and S9—of chemically inactivated cells.

Table 5 summarizes the minimum detectable cell concentrations of various *F. tularensis* strains. As can be seen, the LOD varies from 1 × 10^6^ to 5 × 10^7^ CFU/mL when using the AuNPs2–Fb11 conjugate and from 5 × 10^4^ to 5 × 10^6^ CFU/mL when using the AuNPs2–T143 conjugate. The AuNPs2–T143 conjugate does not agglutinate in the presence of the non-virulent *F. tularensis novicida Utah112* strain, which enables distinguishing virulent and non-virulent *F. tularensis* strains.

Specificity of the assay to heterologous LPS preparations from *Yersinia enterocolitica* 287 and *Salmonella* spp. and cells of *Y. enterocolitica* H-26-04, *Y. pseudotuberculosis* 4320, *Pseudomonas aeruginosa* ATCC27853, and *Brucella abortus* was determined using the AuNPs2–T143 conjugate. Results presented in Figure S10 demonstrated that the AuNPs2–T143 conjugate did not bind to the above-mentioned heterologous antigens.

**Figure 6:**
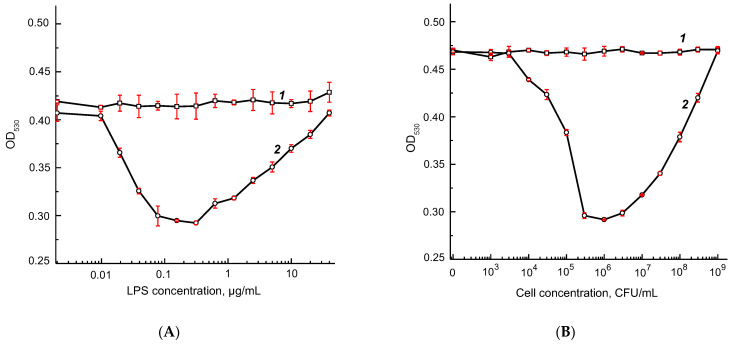
Calibration curves for AuNPs2–T143 agglutination at 530 nm in the presence of different concentrations of *F. tularensis* LPS (**A**) and *F. tularensis* subsp. *holarctica 15 NIIEG* (**B**). Curves 1 and 2 correspond to 1 and 20 min of the reaction. Measurements were made in triplicate.

Calibration dependences for *F. tularensis* LPS (Figure 6A) and *F. tularensis* subsp. *holarctica 15 NIIEG* cells (Figure 6B) were obtained in the chosen optimal assay conditions. The LODs were 40 ng/mL for LPS and 3 × 10^4^ CFU/mL for *F. tularensis* cells, and the detection accuracy (RSD) was 2.5–4.5%.

The determination of *F. tularensis* cells was carried out in natural water from a river, lake, and spring located in the Moscow region. Solutions of *F. tularensis* cells and the AuNPs2–T143 conjugate were prepared in water samples. Triton X-100 (0.05%) was added to prevent non-specific binding. Table 6 summarizes the results of testing of natural and tap water samples spiked with *F. tularensis* subsp. *holarctica 15 NIIEG* after 1 and 20 min of the agglutination reaction. The obtained results demonstrate that the OD_530_ values in the natural water samples differed from the calibration curves by 11.5–30.4% depending on the type of matrix without significant changes in the LODs.

## 4. Conclusions

The test system has been developed for the rapid determination of LPS and *F. tularensis* cells in water samples based on the agglutination of a conjugate of specific antibodies with AuNPs. The LODs of LPS and *F. tularensis* subsp. *holarctica 15 NIIEG* were 40 ng/mL and 3 × 10^4^ CFU/mL, respectively. The determination accuracy was 2.5–4.5%, the assay duration was 20 min. The test system was successfully tested on samples of natural and tap water. The assay is comparable with the known colorimetric nanoparticles-based agglutination tests for microorganisms detection in sensitivity and may be implemented within a short time, determining efficiency of the proposed testing for wide screening control.

## Figures and Tables

**Table 1:** Characteristics of AuNPs.

Preparation	Diameter (TEM Data), nm	Ellipticity	Diameter (DLS Data), nm	Polydispersity Index	Spectral Peak, nm	Diameter (Spectral Data), nm
AuNPs1	26.6 ± 1.6	1.1 ± 0.14	26.8	0.236	523	26
AuNPs2	33.7 ± 2.3	1.2 ± 0.17	36.8	0.266	526	34
AuNPs3	41.8 ± 4.2	1.3 ± 0.27	41.7	0.286	530	42

**Table 2:** Parameters of AuNPs1, AuNPs2, and AuNPs3 and the calculated amount of MAbs for monolayer formation.

Preparation	Diameter of AuNPs, nm	AuNPs Surface Area, nm^2^	IgG Number per One Particle (Monolayer), pcs	[AuNPs], pcs/mL	[IgG] Monolayer, pcs/mL	[IgG] Monolayer, μg/mL
AuNPs1	26	2123	85	0.327 × 10^12^	27.8 ×·10^12^	6.55
AuNPs2	34	3630	145	0.146 ×·10^12^	21.17 ×·10^12^	5.12
AuNPs3	42	5540	222	0.077 ×·10^12^	17.17 × 10^12^	4.16

**Table 3:** Spectrophotometric characterization of AuNPs–MAbs conjugates.

Preparation	Spectral Peak, nm	Diameter, nm (Spectral Data)
AuNPs1	523	26
AuNPs1–Fb11	526	34
AuNPs1–T143	526	34
AuNPs2	526	34
AuNPs2–Fb11	530	42
AuNPs2–T143	530	42
AuNPs3	530	42
AuNPs3–Fb11	532	48
AuNPs3–T143	532	48

**Table 4:** Changes in OD over time for the AuNPs–Fb11 (LPS concentration is 10 µg/mL) and AuNPs–T143 (LPS concentration is 0.3 µg/mL) conjugates.

AuNPs–MAbs Conjugate	Wavelength, nm	Change in OD at Wavelengths of Absorption Maxima, %			
		Reaction Time, min			
		1	10	20	30
AuNPs1–Fb11	526	0	17.4	43.0	100.0
AuNPs1–T143	526	0	23.5	73.0	100.0
AuNPs2–Fb11	530	0	47.7	77.9	100.0
AuNPs2–T143	530	0	68.6	88.9	100.0
AuNPs3–Fb11	532	0	30.6	65.0	100.0
AuNPs3–T143	532	0	69.5	88.8	100.0

**Table 5:** Minimum detectable concentrations of *F. tularensis* cells in the agglutination reaction with AuNPs2–Fb11 and AuNPs2–T143 conjugates.

*F. tularensis* subsp.	Method of Inactivation	Minimum Detectable Concentrations, CFU/mL	
		AuNPs2–Fb11	AuNPs2–T143
*F. tularensis*, subsp. *holarctica 15 NIIEG*	thermal	5 × 10^6^	5 × 10^4^
	chemical	5 × 10^6^	5 × 10^5^
*F. tularensis*, subsp. *holarctica 503*	thermal	1 × 10^6^	5 × 10^5^
	chemical	1 × 10^7^	5 × 10^4^
*F. tularensis*, subsp. *mediasiatica 120*	thermal	1 × 10^7^	5 × 10^5^
	chemical	1 × 10^7^	5 × 10^5^
*F. tularensis*, subsp. *tularensis Schu*	thermal	5 × 10^6^	5 × 10^6^
	chemical	5 × 10^6^	5 × 10^5^
*F. tularensis*, subsp. *tularensis* A-*Cole B-399*	thermal	1 × 10^7^	5 × 10^5^
*F. tularensis*, subsp. *miura*	chemical	5 × 10^7^	1 × 10^6^
*F. tularensis*, subsp. *novicida Utah112*	thermal	5 × 10^6^	no
	chemical	5 × 10^7^	no

**Table 6:** OD_530_ values in water samples spiked with *F. tularensis* subsp. *holarctica 15 NIIEG* cells at concentrations of 10^8^, 10^7^, 10^6^, and 10^5^ CFU/mL after 1 and 20 min of the agglutination reaction.

Water Sample	Cell Concentration, CFU/mL	OD_530_ of the Reaction Mixture, Optical Units		
		Calibration Curve		Sample
		1 min	20 min	20 min
Sample 1 (river water)	10^8^	0.468 ± 0.0031	0.379 ± 0.005	0.424 ± 0.005
	10^7^	0.467 ± 0.0006	0.318 ± 0.0007	0.388 ± 0.001
	10^6^	0.469 ± 0.0052	0.292 ± 0.0003	0.312 ± 0.003
	10^5^	0.468 ± 0.0044	0.383 ± 0.0032	0.412 ± 0.0005
Sample 2 (lake water)	10^8^	0.468 ± 0.0031	0.377 ± 0.025	0.435 ± 0.0015
	10^7^	0.467 ± 0.0006	0.318 ± 0.001	0.358 ± 0.0015
	10^6^	0.466 ± 0.0045	0.294 ± 0.003	0.346 ± 0.0015
	10^5^	0.468 ± 0.0024	0.384 ± 0.0022	0.418 ± 0.0025
Sample 3 (spring water)	10^8^	0.468 ± 0.0031	0.378 ± 0.005	0.424 ± 0.0015
	10^7^	0.467 ± 0.0022	0.319 ± 0.0012	0.382 ± 0.0015
	10^6^	0.466 ± 0.0034	0.293 ± 0.0031	0.335 ± 0.003
	10^5^	0.468 ± 0.0024	0.382 ± 0.002	0.425 ± 0.002
Sample 4 (tap water)	10^8^	0.468 ± 0.0031	0.380 ± 0.0015	0.431 ± 0.0005
	10^7^	0.467 ± 0.0012	0.319 ± 0.001	0.378 ± 0.003
	10^6^	0.467 ± 0.0025	0.292 ± 0.0013	0.342 ± 0.0005
	10^5^	0.468 ± 0.0035	0.382 ± 0.002	0.416 ± 0.003

## Data Availability

Data are presented in the article. Initial instrumental output data are available upon request from corresponding author.

## Supplementary Materials

The following supporting information can be downloaded at: https://www.mdpi.com/article/10.3390/mi13122194/s1, Obtaining *F. tularensis* LPS, Production of monoclonal antibodies against *F. tularensis* LPS, Figure S1 Diameter distribution of AuNPs with average diameters of 26.8 (A), 36.8 (B), and 41.7 (C) nm (data of DLS measurements), Figure S2: Absorption spectra of AuNPs1 (A), AuNPs2 (B), and AuNPs3 (C). The red arrows indicate the maximum of the spectra, Figure S3: Diameter distribution of AuNPs–Fb11 conjugates with average diameters of 36.6 (AuNPs1–Fb11; (A)), 43.9 (AuNPs2–Fb11; (B)), and 57.8 (AuNPs3–Fb11; (C)) nm (data of DLS measurements), Figure S4: Diameter distribution of AuNPs–T143 conjugates with average diameters of 30.2 (AuNPs1–T143; (A)), 37.5 (AuNPs2–T143; (B)), and 50.8 (AuNPs3–T143; (C)) nm (data of DLS measurements), Figure S5: Agglutination spectra of the AuNPs2–Fb11 conjugate after 1 (A), 10 (B), 20 (C), and 30 (D) min of the reaction. Curves correspond to 30 (1), 10 (2), 3 (3), 1 (4), 0.3 (5), 0.1 (6), 0.03 (7), and 0 (8) µg/mL *F. tularensis* LPS, Figure S6: Agglutination spectra of the AuNPs2–T143 conjugate after 1 (A), 10 (B), 20 (C), and 30 (D) min of the reaction. Curves correspond to 30 (1), 10 (2), 3 (3), 1 (4), 0.3 (5), 0.1 (6), 0.03 (7), and 0 (8) µg/mL *F. tularensis* LPS, Figure S7: Visual registration of agglutination of AuNPs1–MAbs (1), AuNPs2–MAbs (2), and AuNPs3–MAbs (3) conjugates after 1 (A,C,E) and 30 (B,D,F) min of the reaction. Each reaction was performed in duplicate, Figure S8: Agglutination of AuNPs2–Fb11 conjugates in the presence of chemically inactivated *F. tularensis* subsp. *holarctica 15 NIIEG* (A) and *503* (B), *F. tularensis* subsp. *mediasiatica 120* (C), *F. tularensis* subsp. *tularensis Schu* (D), *F. tularensis* subsp. *miura* (E), and *F. tularensis* subsp. *novicida Utah112* (F) at different concentrations. Curves 1 and 2 correspond to 1 and 20 min of the reaction, Figure S9: Agglutination of AuNPs2–T143 conjugates in the presence of chemically inactivated *F. tularensis* subsp. *holarctica 15 NIIEG* (A) and *503* (B), *F. tularensis* subsp. *mediasiatica 120* (C), *F. tularensis* subsp. *tularensis Schu* (D), *F. tularensis* subsp. *miura* (E), and *F. tularensis* subsp. *novicida Utah112* (F) at different concentrations. Curves 1 and 2 correspond to 1 and 20 min of the reaction, Figure S10: Agglutination spectra of the AuNPs2–T143 conjugate in the presence of *Yersinia enterocolitica H-26-04* cells (A), *Y. pseudotuberculosis 4320* (B), *Pseudomonas aeruginosa ATCC27853* (C), *Brucella abortus* (D) and 10 µg/mL LPS *Salmonella* spp. (E) and *Y. enterocolitica 287* (F) at the concentration of 10^7^ CFU/mL. Curves correspond to 1 (black) and 10 (red) min of the reaction.
