# Supplementary material for: Rapid Detection of Lipopolysaccharide and Whole Cells of Francisella tularensis Based on Agglutination of Antibody-Coated Gold Nanoparticles and Colorimetric Registration
Source: Micromachines (Basel). 2022 Dec 11;13(12):2194. doi: 10.3390/mi13122194 (PMC9784915; doi:10.3390/mi13122194)
Supplement: Supplementary file 1 [file micromachines-13-02194-s001.zip › micromachines-2065316-supplementary.pdf]

Supplementary materials

Rapid detection of lipopolysaccharide and whole cells of *Francisella tularensis* based on agglutination of antibody-coated gold nanoparticles and colorimetric registration

Nadezhda A. Byzova[1], Anatoly V. Zherdev[1], Alexey A. Gorbatov[2], Anton G. Shevyakov[2], Sergey F. Biketov[2], Boris B. Dzantiev[1]

[1] – A.N. Bach Institute of Biochemistry, Research Center of Biotechnology, Russian Academy of Sciences, 119071 Moscow, Russia

[2] – State Research Center for Applied Microbiology and Biotechnology, 142279 Obolensk, Moscow region, Russia

Table of Contents

Table of Figures …………………………………………………………………………. 3

Materials and Methods ……………………………………………………………….. 4

Obtaining *F. tularensis* LPS ………………………………………………………….. 4

Production of monoclonal antibodies against *F. tularensis* LPS ………………………….. 4

Results and Discussion ……………………………………………………………….. 5

Characterization of nanoparticles …………………………………………………….. 5

Characterization of AuNPs–MAbs conjugates ……………………………………………. 7

Agglutination spectra of AuNPs–MAbs conjugates ………………………………………. 9

Visual registration of agglutination of AuNPs–MAbs conjugates ………………………… 11

Agglutination of AuNPs–MAbs conjugates in the presence of cells …………………....... 12

References ……………………………………………………………………………. 15

Table of Figures

Figure S1 ………………………………………………………………………………… 5

Figure S2 ………………………………………………………………………………… 6

Figure S3 ………………………………………………………………………………… 7

Figure S4 ………………………………………………………………………………… 8

Figure S5 ………………………………………………………………………………… 9

Figure S6 ………………………………………………………………………………… 10

Figure S7 ………………………………………………………………………………… 11

Figure S8 ………………………………………………………………………………… 12

Figure S9 ………………………………………………………………………………… 13

Figure S10 ………………………………………………………………………………... 14

Materials and Methods

Obtaining F. tularensis LPS

The *F. tularensis* subsp. *holarctica 15 NIIEG* strain was used to obtain LPS. The isolation of LPS was carried out by the extraction according to [1]. Bacterial cells were heated at 68 °C in a 45% aqueous phenol solution and centrifuged (10,000 g, 30 min). The aqueous phase containing LPS, nucleic acids, and polysaccharides was collected and dialyzed against deionized water for 4 days [2].

Purification of LPS from nucleic acids was carried out using DNase and RNase. LPS solutions were incubated at 37 °C for 3–18 h in the presence of 10 mM Tris-HCl, pH 7.2, 10 mM magnesium sulfate, and 100 µg/mL of DNAse and RNAse. The hydrolysis of nucleic acids was monitored spectrophotometrically and electrophoretically in a 0.7% agarose gel stained with ethidium bromide. Purification of LPS from proteins was carried out using proteinase K. LPS solutions were incubated at 65 °C for 3–5 h in the presence of proteinase K (50 µg/mL) and dialyzed against deionized water for 12 h followed by fourfold centrifugation for 2 h at 100,000 g. The content of nucleic acids in the supernatant was controlled by a decrease in optical density (OD) at 260 nm. The purified LPS preparations were lyophilized.

Production of monoclonal antibodies against F. tularensis LPS

To obtain monoclonal antibodies (MAbs), hybridomas were cultured in a CO_2 incubator in PRMI-1640 medium supplemented with 10% heat-inactivated fetal bovine serum at 37 °C in the presence of 5% CO_2. After 4-6 days of growth, hybridoma cells were concentrated by centrifugation at 500 g for 5 min and resuspended in sterile 0.9% NaCl solution to a final concentration of 10^6 cells/mL. Then, 1 mL of this mixture was intraperitoneally injected into BALB/c mice. After 7–10 days, ascitic fluid containing MAbs was collected. Cells were removed from the ascitic fluid by centrifugation at 1000 g for 15 min. The supernatant was mixed with 0.1 M phosphate buffer, pH 8.6, in a ratio of 1:4 and filtered through a filter with a pore diameter of 0.22 µm. MAbs were purified by affinity chromatography on a protein-A-sepharose column. MAbs were eluted with 0.1 M citrate buffer, pH 3.0, at a rate of 0.5 mL/min and then with 50 mM potassium phosphate buffer, pH 7.4, containing 0.1M NaCl (PBS), on a Sephadex G-25 column. The concentration of immunoglobulins was determined spectrophotometrically.

Results and Discussion

Characterization of nanoparticles

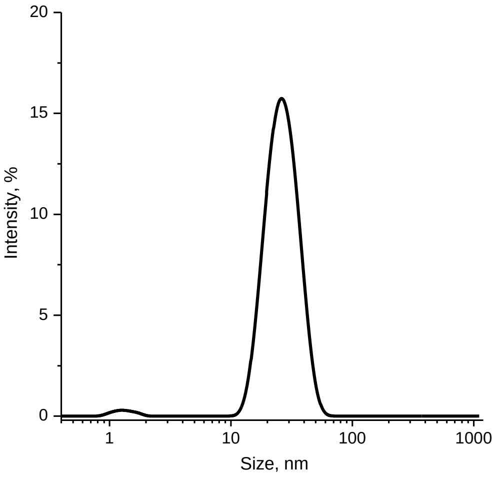

(A)

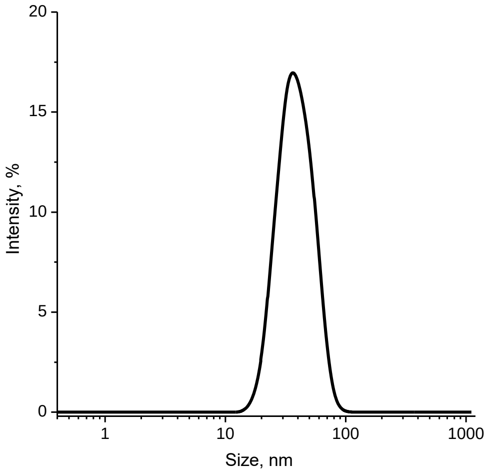

(B)

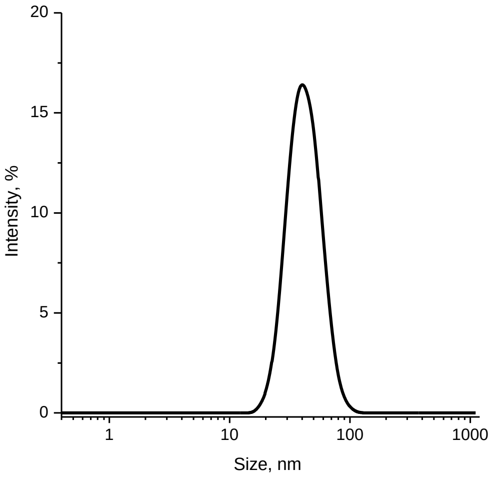

(C)

Figure S1. Diameter distribution of AuNPs with average diameters of 26.8 (A), 36.8 (B), and 41.7 (C) nm (data of DLS measurements).

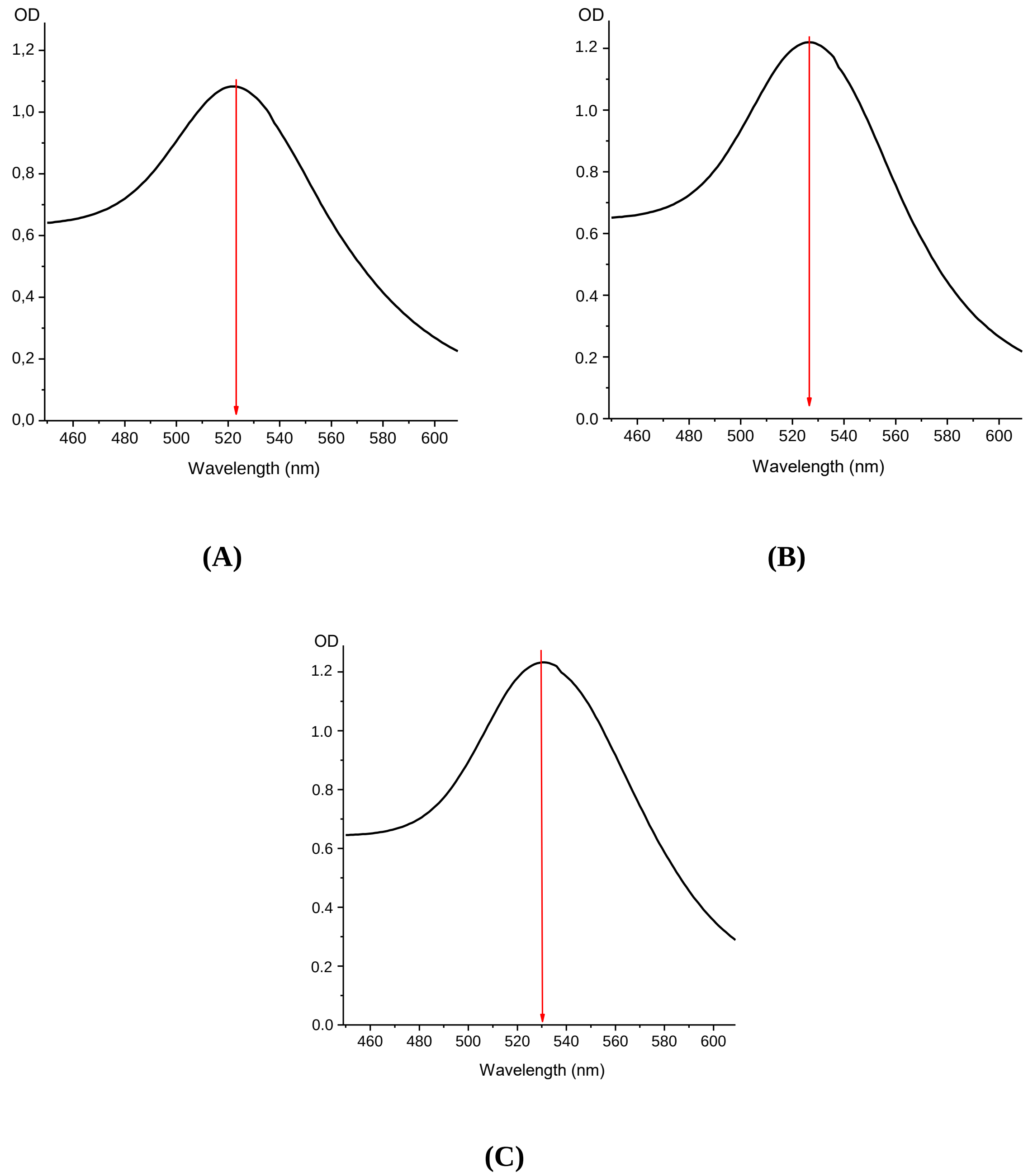

Figure S2. Absorption spectra of AuNPs1 (A), AuNPs2 (B), and AuNPs3 (C). The red arrows indicate the maximum of the spectra.

Characterization of AuNPs–MAbs conjugates

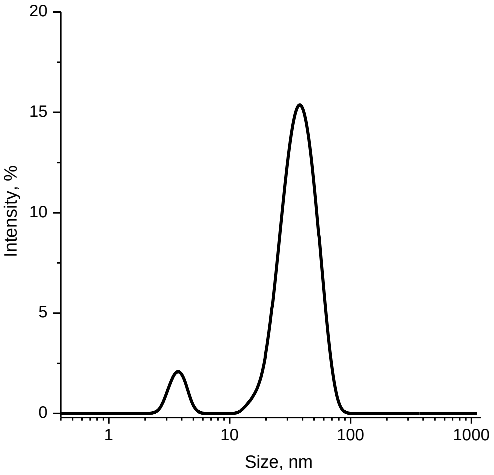

(A)

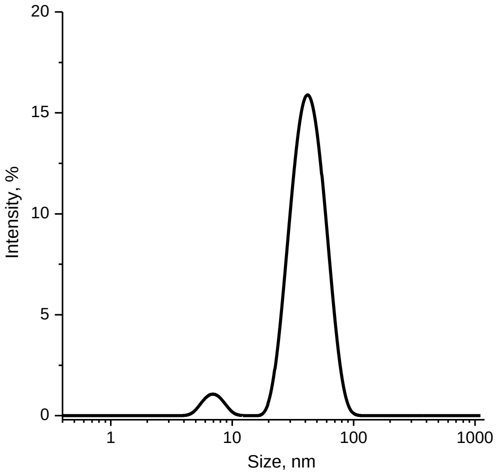

(B)

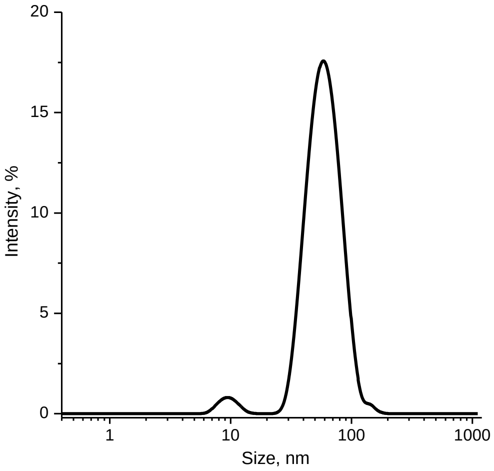

(C)

Figure S3. Diameter distribution of AuNPs–Fb11 conjugates with average diameters of 36.6 (AuNPs1–Fb11; A), 43.9 (AuNPs2–Fb11; B), and 57.8 (AuNPs3–Fb11; C) nm (data of DLS measurements).

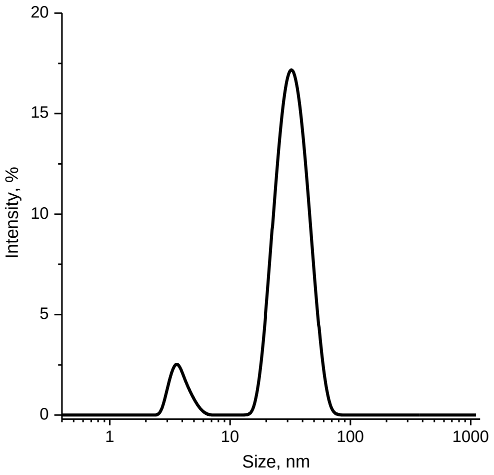

(A)

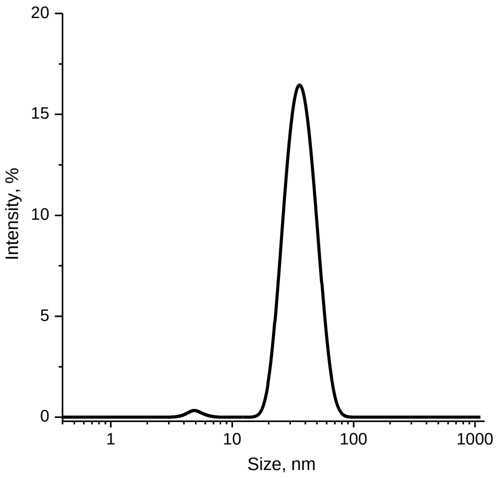

(B)

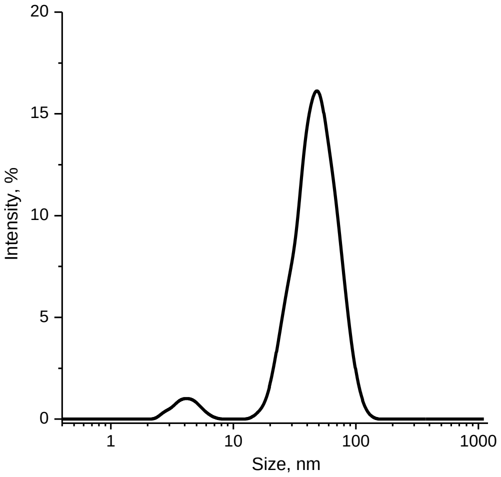

(C)

Figure S4. Diameter distribution of AuNPs–T143 conjugates with average diameters of 30.2 (AuNPs1–T143; A), 37.5 (AuNPs2– T143; B), and 50.8 (AuNPs3–T143; C) nm (data of DLS measurements).

Agglutination spectra of AuNPs–MAbs conjugates

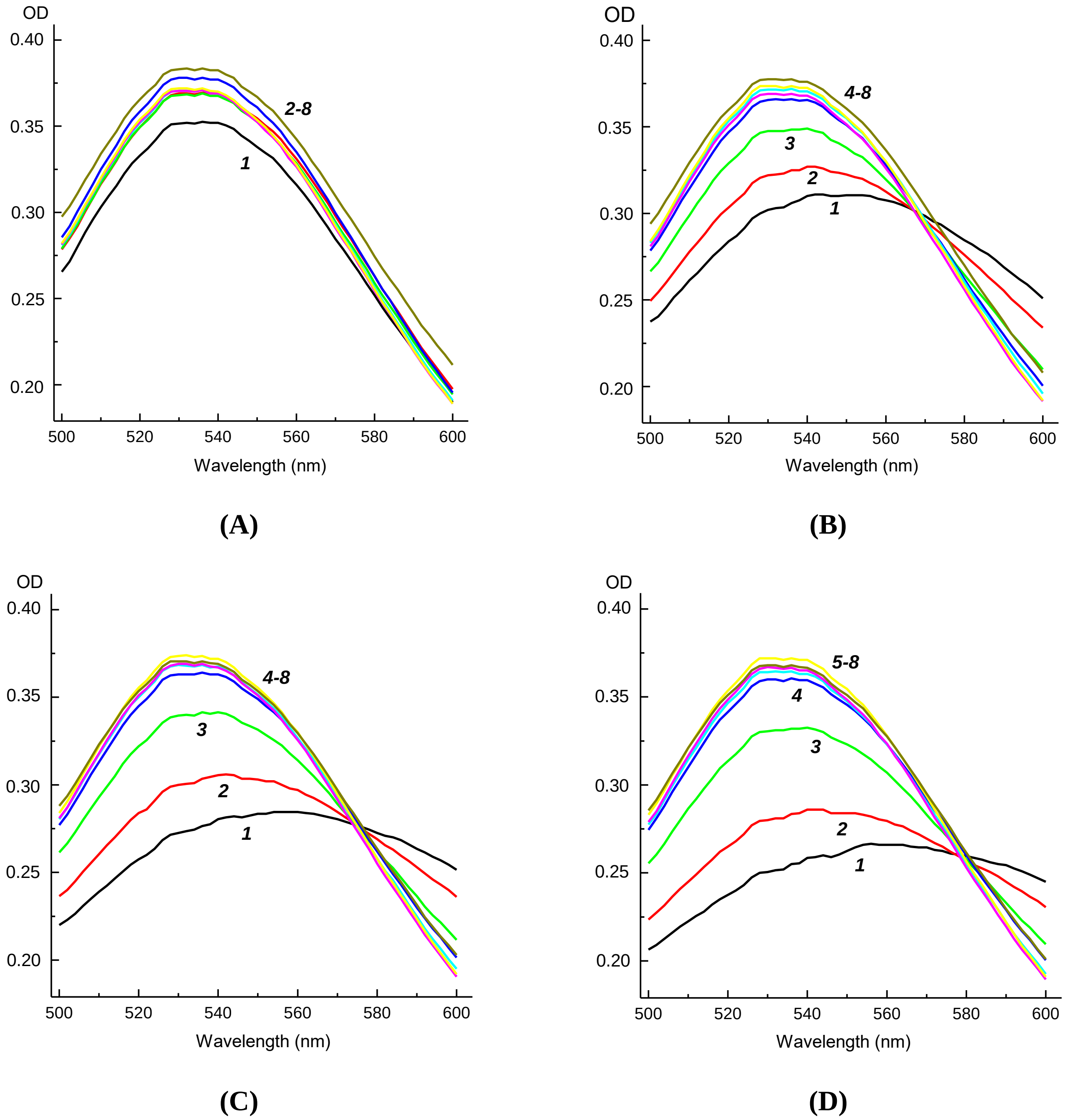

Figure S5. Agglutination spectra of the AuNPs2–Fb11 conjugate after 1 (A), 10 (B), 20 (C), and 30 (D) min of the reaction. Curves correspond to 30 (1), 10 (2), 3 (3), 1 (4), 0.3 (5), 0.1 (6), 0.03 (7), and 0 (8) µg/mL *F. tularensis* LPS.

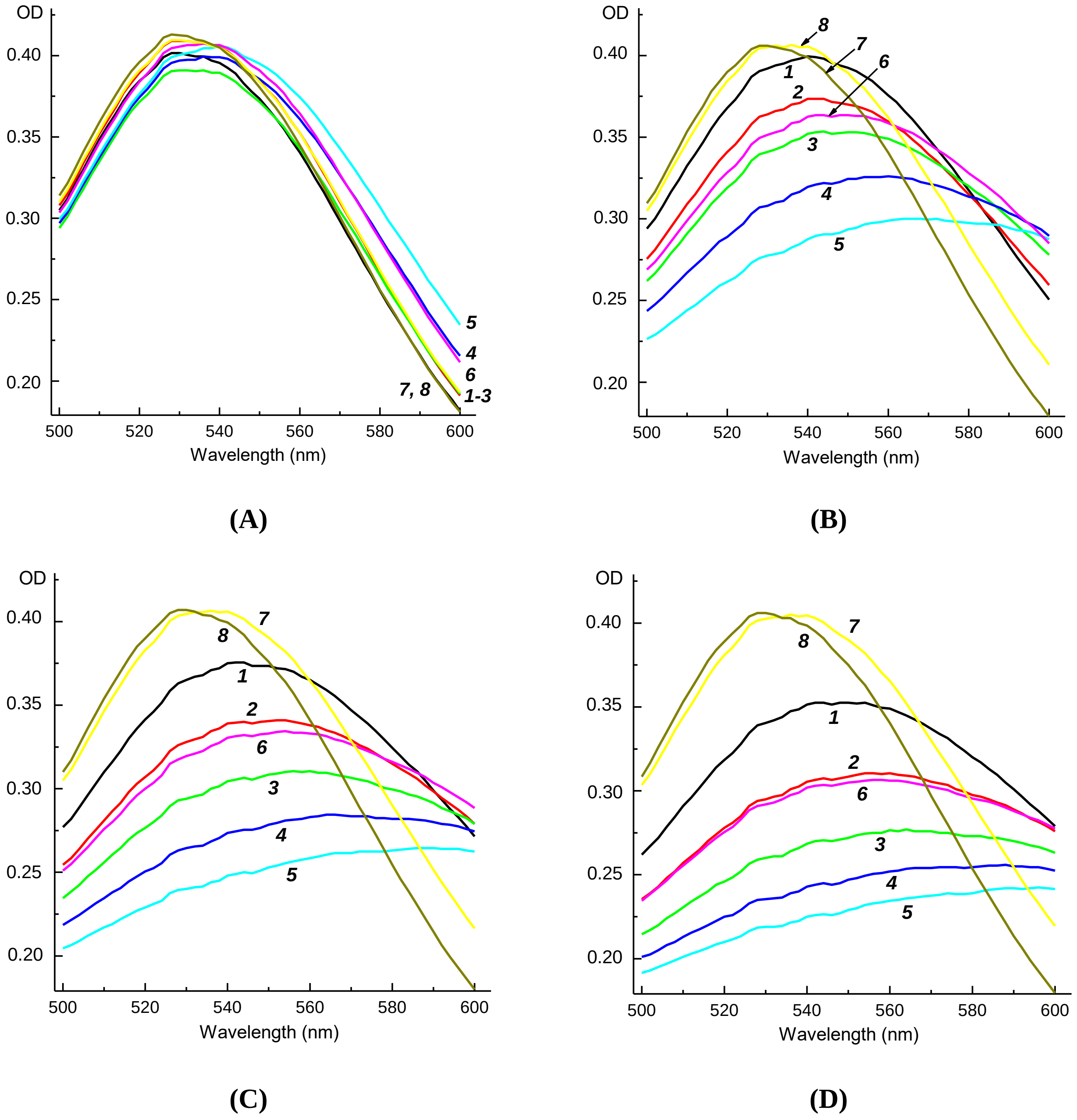

Figure S6. Agglutination spectra of the AuNPs2–T143 conjugate after 1 (A), 10 (B), 20 (C), and 30 (D) min of the reaction. Curves correspond to 30 (1), 10 (2), 3 (3), 1 (4), 0.3 (5), 0.1 (6), 0.03 (7), and 0 (8) μg/mL *F. tularensis* LPS.

Visual registration of agglutination of AuNPs–MAbs conjugates

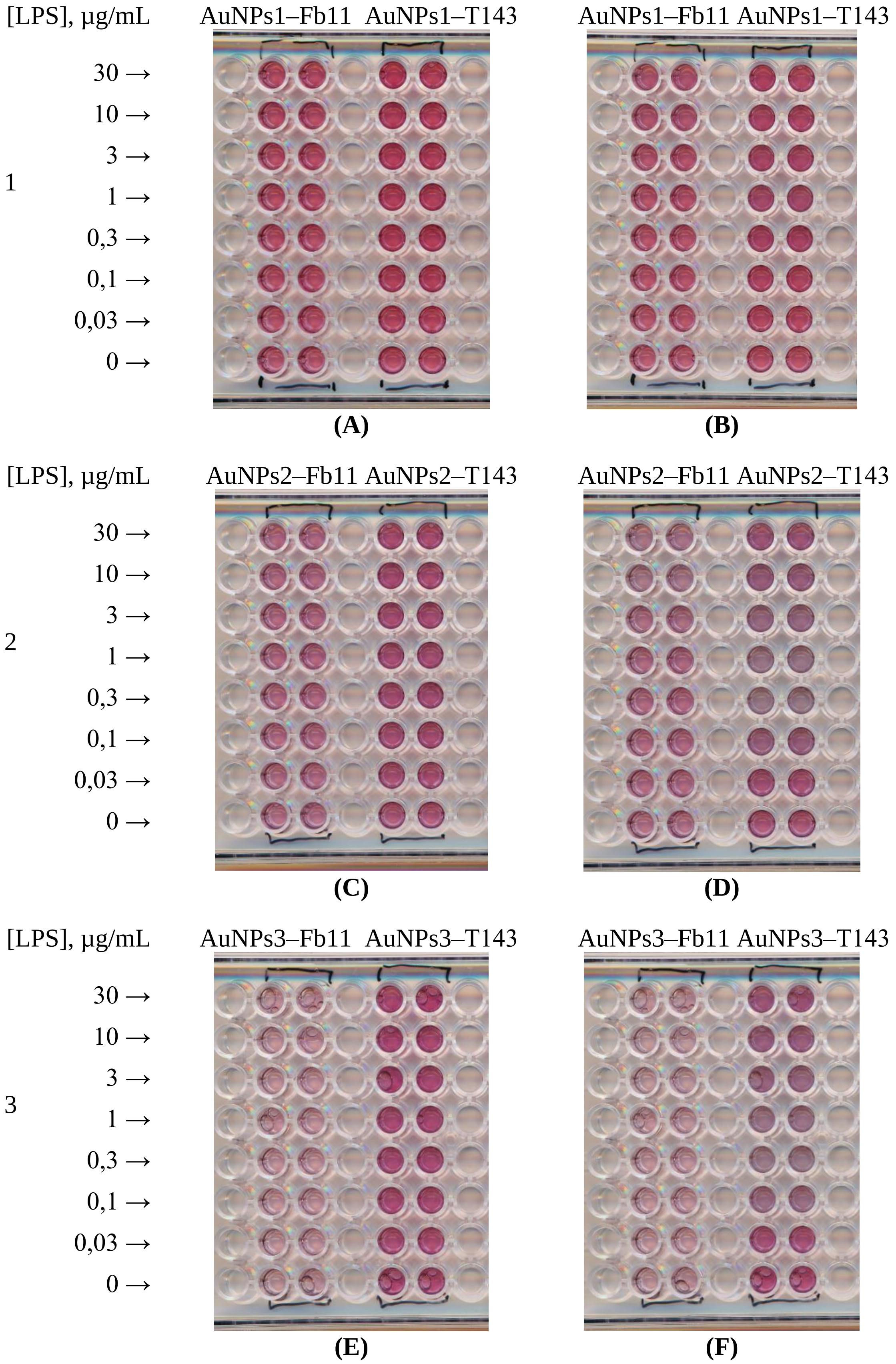

Figure S7. Visual registration of agglutination of AuNPs1–MAbs (1), AuNPs2–MAbs (2), and AuNPs3–MAbs (3) conjugates after 1 (A, C, E) and 30 (B, D, F) min of the reaction. Each reaction was performed in duplicate.

Agglutination of AuNPs–MAbs conjugates in the presence of cells

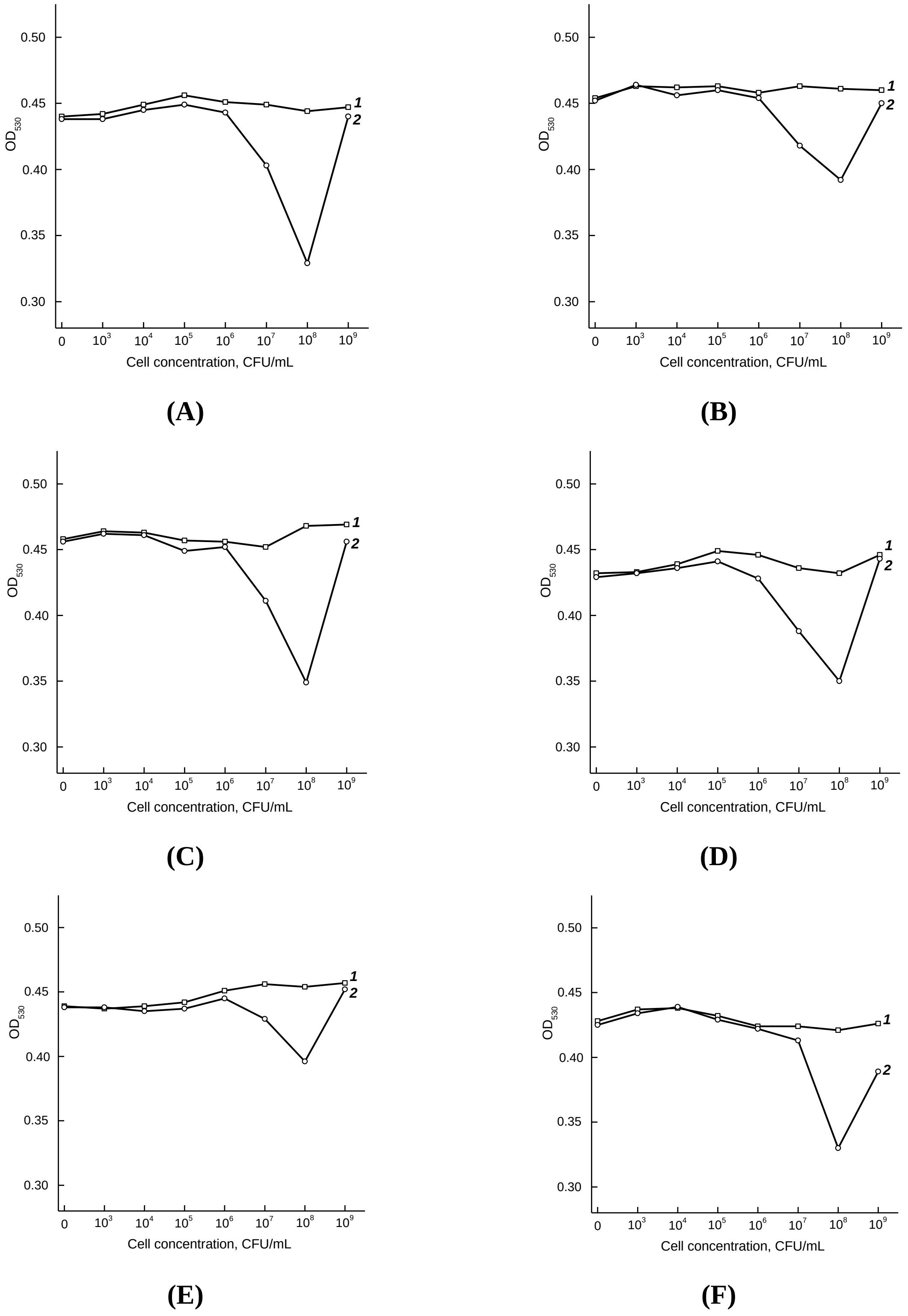

Figure S8. Agglutination of AuNPs2–Fb11 conjugates in the presence of chemically inactivated *F. tularensis* subsp. *holarctica 15 NIIEG* (A) and *503* (B), *F. tularensis* subsp. *mediasiatica 120* (C), *F. tularensis* subsp. *tularensis Schu* (D), *F. tularensis* subsp. *miura* (E), and *F. tularensis* subsp. *novicida Utah112* (F) at different concentrations Curves 1 and 2 correspond to 1 and 20 min of the reaction.

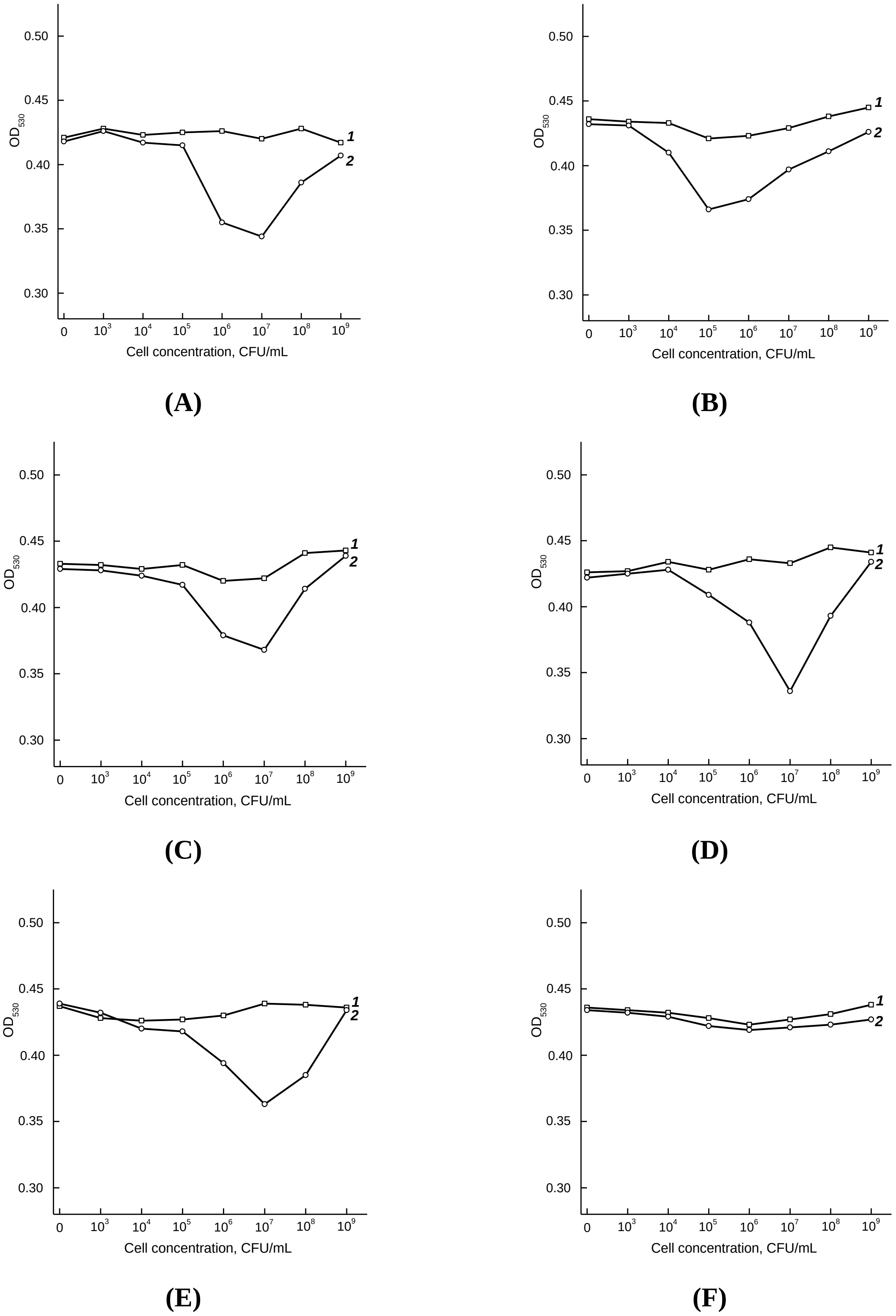

Figure S9. Agglutination of AuNPs2–T143 conjugates in the presence of chemically inactivated *F. tularensis* subsp. *holarctica 15 NIIEG* (A) and *503* (B), *F. tularensis* subsp. *mediasiatica 120* (C), *F. tularensis* subsp. *tularensis Schu* (D), *F. tularensis* subsp. *miura* (E), and *F. tularensis* subsp. *novicida Utah112* (F) at different concentrations. Curves 1 and 2 correspond to 1 and 20 min of the reaction.

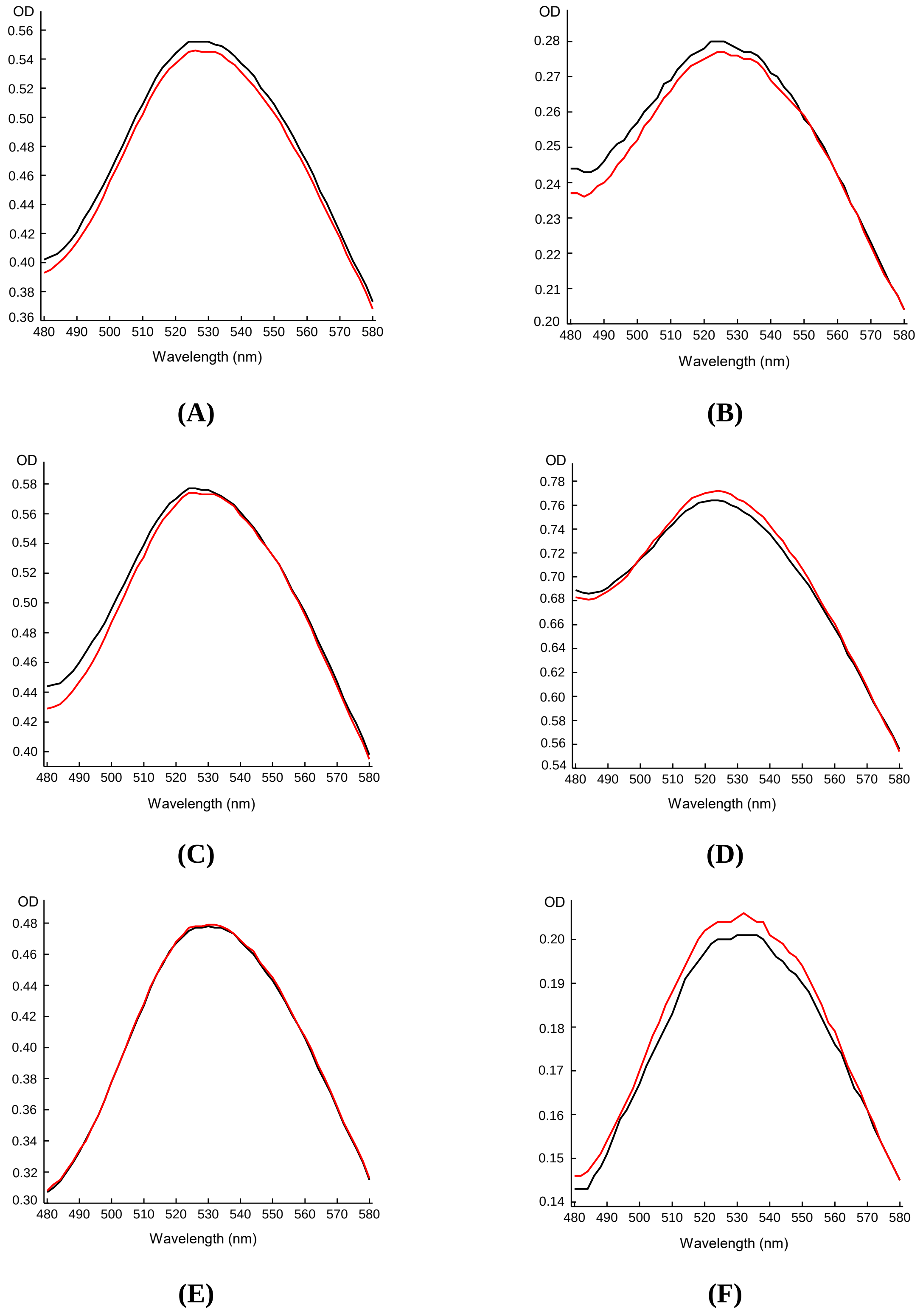

Figure S10. Agglutination spectra of the AuNPs2–T143 conjugate in the presence of *Yersinia enterocolitica H-26-04* (A), *Y. pseudotuberculosis 4320* (B), *Pseudomonas aeruginosa ATCC27853* (C), *Brucella abortus* (D) cells at the concentration of 10^7 CFU/mL and 10 µg/mL LPS *Salmonella spp*. (E) and *Y. enterocolitica 287* (F). Curves correspond to 1 (black) and 10 (red) min of the reaction.

References

1. Westphal, O.; Jann, K. Bacterial lipopolysaccharides. Extraction with phenol-water and further applications of the procedure. *Meth. Carbohydrate Chem*. **1965**, *5*, 83–91.
2. Wetmur, J.G.; Davidson, N.J. Kinetics of renaturation of DNA. *J. Mol. Biol*. **1968**, *31*, 349–370, DOI: 10.1016/0022-2836(68)90414.
